# Residual Efficacy of Novaluron Applied on Concrete, Metal, and Wood for the Control of Stored Product Coleopteran Pests

**DOI:** 10.3390/insects12010007

**Published:** 2020-12-25

**Authors:** Muhammad Yasir, Richard W. Mankin, Mansoor ul Hasan, Muhammad Sagheer

**Affiliations:** 1 Department of Entomology, University of Agriculture, Faisalabad 38000, Pakistan; yasiruca@gmail.com (M.Y.); mansoorsahi2000@yahoo.com (M.u.H.); sagheersharif@yahoo.com (M.S.); 2 Agricultural Research Service Center for Medical, Agricultural and Veterinary Entomology (CMAVE), United States Department of Agriculture, Gainesville, FL 32608, USA

**Keywords:** chitin synthesis inhibitor, insect growth regulator, surface treatment

## Abstract

**Simple Summary:**

Insect pests of stored commodities cause harm not only to bulk grains but also to many value-added food products in mills, processing plants, and other facilities where these products are stored. In this study, the residual efficacy of an Insect Growth Regulator (IGR), novaluron, was evaluated under laboratory conditions against larvae of three stored product insects, *Oryzaephilus surinamensis* (L.), *Tribolium* *castaneum* (Herbst), and *Trogoderma granarium* Everts, on concrete, metal, and wood surfaces to which IGRs are typically applied for pest control in such facilities. Statistically significant reductions in emergence percentages of adults compared to a distilled water control occurred for up to 12 weeks when novaluron was sprayed on concrete, metal, and wood at rates between 0.053 and 0.209 mg/m^2^, the highest of which induces 100% mortality at 0 weeks after treatment. Residual efficacy decreased with increasing time after treatment due to degradation and sorption of novaluron into the surfaces. Novaluron residues were most persistent on metal and least persistent on wood surfaces. Knowledge of novaluron residual efficacy on storage facility surfaces can be helpful guidance for timing of postharvest insect pest management treatments as the costs of commonly used pesticides increase along with insect resistance to such pesticides.

**Abstract:**

The residual efficacy of novaluron on concrete, metal and wood was evaluated against last-instar larvae of *Oryzaephilus surinamensis* (L.), *Tribolium* *castaneum* (Herbst), and *Trogoderma granarium* Everts. The larvae and food provided for survival were exposed to surfaces pretreated at rates of 0.053, to 0.209 mg/m^2^ and bioassays were conducted from 0- to 16-weeks post-treatment. Percentage emergence of adults was recorded after 30 days (d). On all surfaces at week 0, no *O. surinamensis* or *T.* *castaneum* adults emerged, and ≤3.3% emergence of *T. granarium* was found at 0.209 mg/m^2^. Novaluron significantly reduced the percentage emergence of adults of the three species compared to a distilled water control for the first 12 weeks on all the tested surfaces as the residual efficacy declined at a low rate during initial weeks and then at a high rate in the final weeks of the 16-week study. Reductions to emergence were most persistent on metal surfaces, with mean percentages of adult emergence of ≤18.3 in week 12, followed by concrete (≤32.5) and wood (≤45.0) for all species at novaluron application rates of 0.209 mg/m^2^. For >4-weeks protection, higher application rates would be needed to avoid buildup of pest populations and reductions in profitability. Such results can be helpful for the management of *O. surinamensis*, *T.* *castaneum*, and *T. granarium* as the costs of commonly used insecticides against postharvest insect pests and the resistance of these insects to the pesticides gradually increase in mills, warehouses, and food storage facilities.

## 1. Introduction

Currently, the main strategies for the control of stored product insect pests are based on fumigants (primarily phosphine) and residual insecticides that are applied as surface treatment or as grain protectants in food storage facilities [1,2]. However, most of these treatments leave toxic residues, and their frequent use had led to the development of resistance in stored product insects to the most-used insecticides [3,4,5,6,7]. In recent years, research has been conducted on non-chemical control methods, e.g., botanicals, pheromones, or hermetic storage [8,9,10,11], as well as alternative chemical insecticides with low toxicity to mammals and humans [12,13,14]. Need remains, however, for further evaluation of methods to apply alternative reduced risk insecticides in ways that provide effective, nontoxic control of important stored-product insect pest.

Insect growth regulators (IGRs) possess great pest-control potential and their adoption in the food industry has been widely considered [12,15,16,17], particularly for control of stored product insects [18,19,20,21,22,23,24,25,26]. A prominent use of IGRS is to spray them onto uncovered metal, wood, or concrete surfaces, as in a study by Boukouvala and Kavallieratos [27] that evaluated six different insecticides on concrete for control of *Trogoderma granarium* Everts (Coleoptera: Dermestidae), including pyriproxyfen, sprayed at a rate of 2.3 mg/m^2^, *s*-methoprene at 3 mg/m^2^, and deltamethrin at 1100 mg/m^2^. Surface treatment approaches generally are less costly and more convenient, and they reduce levels of residues on food commodities compared to direct application [13].

One of the insect growth regulators, novaluron, is a chitin synthesis inhibitor that belongs to the benzoylphenyl urea insecticides. It interferes with developmental processes in immature insects, causing abnormal endocuticular deposition and abortive molting [26,28,29,30]. It has provided control of larval beetle pests, such as *Leptinotarsa decemlineata* (Coleoptera: Chrysomelidae), when applied to foliage at rates of 2.5–7.5 mg/m^2^ or through direct contact with sprays of 10–100 parts per million (ppm) [31]. Novaluron ingested by adults often can be transferred transovarially to eggs, thereby reducing populations of some economically important pest species [32,33,34] and it has low toxicity to several important parasitoids that are used as natural enemies of insect pests [35]. Novaluron is beginning to receive attention for the potential management of stored product insect pests [14,31,32,36,37].

Methods for assessing IGRs against stored product insects include exposing the late instar larvae on treated surfaces or exposing adults with food on treated surfaces and assessing their progeny production [36,37]. The porosity of the surface to be treated is a major factor affecting the toxicity and residual efficacy of surface treatments [38]; generally, insecticide residues are more persistent on non-porous surfaces such as metal or tile and less persistent on porous surfaces such as wood or concrete [36,39].

Several studies have been conducted to explore the residual efficacy of novaluron and other insect growth regulators against stored product insect pests. The time over which novaluron dissipates by 90% at 25 °C in soil is approximately 100 day (d) [28]; consequently, it may have good longevity in dry, sun-protected storage facility surfaces. Arthur and Fontenot [36] exposed late-stage larvae of *Tribolium castaneum* (Herbst) (Coleoptera: Tenebrionidae) and *T. confusum* Jacquelin duVal (Coleoptera: Tenebrionidae) to concrete surfaces treated with novaluron or methoprene. Novaluron applied either 0 or 8 weeks before larval exposure at an application rate of 30 mg active ingredient (AI)/m^2^ resulted in 0% morphologically normal adult *T. castaneum* 0 weeks after treatment and 17.5% normal adults 8 weeks after treatment [36]. In another study, the residual efficacy of methoprene was considered for several stored product insect pests alone or in combination with aeration treatments [40]. A combination treatment of novaluron and pyriproxyfen was explored by exposing eggs, larvae, or adult of *T. castaneum*, *Trogoderma variabile* Ballion (Coleoptera: Dermestidae) and *Dermestes maculatus* (Coleoptera: Dermestidae) to treated concrete surfaces 0–16 weeks after treatment with the labeled application rate [14]. No *T. castaneum* eggs or larvae exposed to the combination treatment emerged as adults.

Novaluron surface treatments against *T. castaneum* have been evaluated only on concrete surfaces and have not been explored in comparisons with *T. granarium* and *Oryzaephilus surinamensis* (L.), (Coleoptera: Silvanidae), which also are economically important pests of stored products in warehouses and food processing facilities. The objective of this study was to evaluate the residual efficacy of novaluron applied to concrete, metal, and wood against *O. surinamensis*, *T. castaneum*, and *T. granarium* and consider future strategies in which novaluron may have applications to control these and other important stored product insect pests. A goal was to identify minimum levels of effective application rates to avoid environmental problems caused by insecticide residues.

## 2. Materials and Methods

### 2.1. Insects

For this study, cultures of *O. surinamensis*, *T. castaneum*, and *T. granarium* were collected from household granaries, grain markets and stores of the Punjab food department in Faisalabad, Pakistan. Previously, it has been observed that considerable variability exists in resistance to pesticides in field insects from different regions of Pakistan [41] and India [42]. Consequently, the heterogeneity of the collections in this study primarily represents that of the Faisalabad region. The collected insect species were reared separately in sterilized glass jars on a 12:12 (Light:Dark) cycle at 30 ± 2 °C and 65 ± 5% relative humidity to obtain a uniformly aged first generation (F_1_). The culture medium was sterilized wheat flour, whole wheat grains and cracked wheat grains for rearing of *T. castaneum*, *T. granarium*, and *O. surinamensis*, respectively.

### 2.2. Preparation of Surfaces and Treatments

Concrete, metal, and wood surfaces were prepared within disposable plastic Petri dishes (surface area, 63.6 cm^2^). The metal surfaces were prepared using galvanized steel cut with machinery to the Petri dish dimensions. The wood surfaces were prepared by cutting plywood rounds that fit into the bottom of the Petri dish. The metal and wood surfaces were sealed at the edges with silicone sealant to prevent the insects from moving to the underside of the surface. The concrete surface was prepared by mixing 1 kg of cement (Maple Leaf Cement, Lahore, Pakistan) with 260 mL of warm water, similarly to methods in [13,36,43,44]. Approximately 20 g of cement paste was placed at the bottom of each Petri dish. The dishes were air-dried for 1 d before use.

A commercial formulation of novaluron, 10% EC (Uniron^®^), ICI LTD., Karachi, Pakistan, was used to prepare the stock solution. Treatments were formulated by mixing 4 microliters of novaluron into 100 mL distilled water in a volumetric flask to make a 4-ppm solution. The 2- and 1-ppm formulations were prepared from this formulation by transferring 50 mL of the 4 ppm solution into a second volumetric flask and adding 50 mL distilled water, then transferring 50 mL from this 2 ppm solution into a third volumetric flask and adding 50 mL distilled water. A micro-pipette sprayer (Biorays, Faisalabad, Pakistan) was used to treat the 63.6 cm^2^ surface of each Petri dish with 0.30 mL of the selected formulation, similarly to methods in [14]. This resulted in application rates of 0.209, 0.1045, and 0.053 mg/m^2^, respectively, for the 4, 2, and 1 ppm concentrations. In preliminary trials, each of these application rates had resulted in ≤3.3% adult emergence from late-instar larvae of all three insect species.

For control treatments, the Petri dish surfaces were treated with sprays of 0.30 mL distilled water. All treated Petri dishes were kept at 25 ± 2 °C, 65 ± 5% Relative Humidity and continuous darkness to dry for 24 h.

### 2.3. Bioassays

For all bioassays, 30 last-instar larvae (three weeks old) of each tested species were introduced into a treated Petri dish after it had been held for a specific post-exposure period. Immediately before the introduction of insects, the food (5 cracked wheat grains for *O. surinamensis*, 0.5 g wheat flour for *T. castaneum*, and 5 whole wheat grains for *T. granarium*,) was added to avoid mortality through starvation. The insects were maintained at 28 ± 2 °C, 65 ± 5% RH and continuous darkness. The number of adults was recorded after 30 days. There were four replicates in bioassays of each concentration (0, 1, 2, and 4 ppm), three surfaces (concrete, metal, and wood), and three insect species (*O. surinamensis*, *T. castaneum*, and *T. granarium*). Bioassays were conducted at one of 6 different times: 0, 2, 4, 8, 12, and 16 weeks after the Petri dishes were treated.

### 2.4. Statistical Analysis

The experiment used a Completely Randomized Design with four replications of each treatment. Percentage emergence of adults was statistically analyzed separately for each species and each week after treatment exposure by using the R-software agricolae package (version 3.5.2: Statistical Procedures for Agricultural Research) [45] to perform analysis of variance (ANOVA). For each species and exposure period, a separate ANOVA was performed to estimate statistical significance in comparisons of differences in percentage emergence among treatments of different concentrations on a specified surface, or in comparisons of differences in percentage emergence among treatments of different surfaces with a specified concentration. The means of percent adult emergence from the different treatments were compared by Tukey–Kramer HSD test at the 0.05 level of significance [46].

## 3. Results

The ANOVA for main effects and their interactions were significant for each species (Table 1).

**Figure 1:**
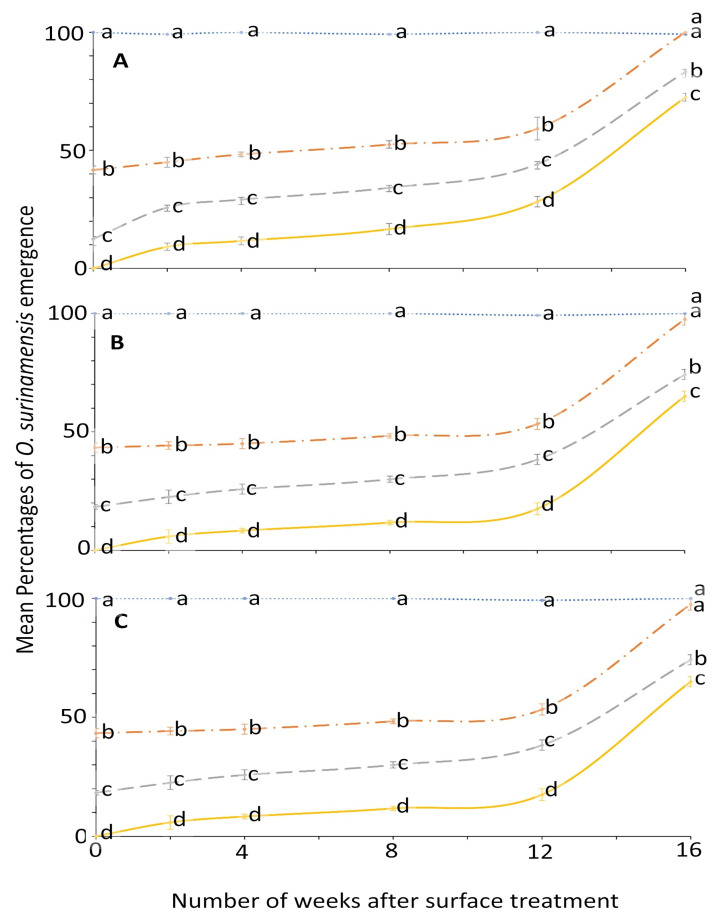
Mean percentages of adults emerging from last-instar *O. surinamensis* after exposure to novaluron applied 0–16 weeks previously at concentrations of 4- (solid line), 2- (dashed line), 1- (dashed-dotted line), or 0-ppm (dotted line) onto different surfaces: (**A**) concrete; (**B**) metal; or (**C**) wood. In comparisons among mean emergence percentages from different concentrations on a given week after application to a specific surface, designated a–d above, means with the same letter are not significantly different.

The effect of concentration on *O. surinamensis* percentage adult emergence was statistically significant from week 0 to 12; later, it became variable in week 16 (Figure 1).

**Figure 2:**
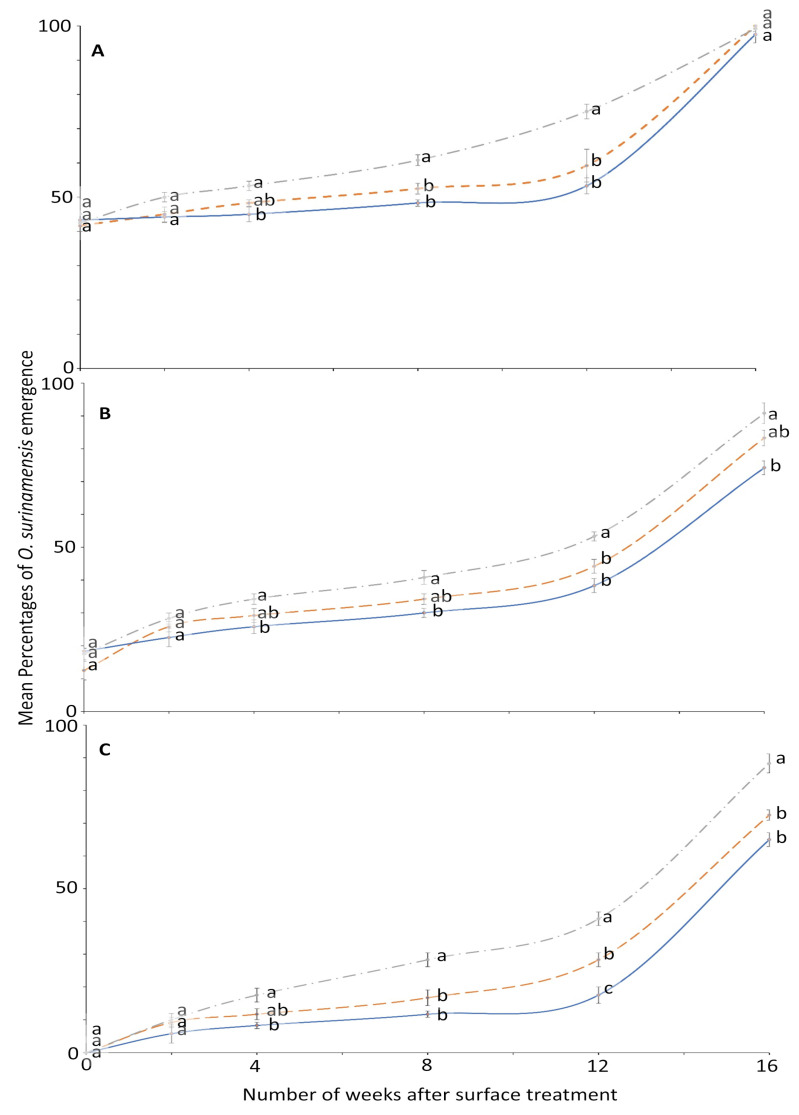
Mean percentages of adults emerging from last-instar *O. surinamensis* after exposure to novaluron applied 0–16 weeks previously to different surfaces: concrete (dashed line); metal, (solid line); or wood (dashed-dotted line) at concentrations of (**A**) 1-, (**B**) 2-, or (**C**) 4-ppm. In comparisons among mean emergence percentages from different surfaces on a given week after application at a specified concentration, designated a–d above, percentages with the same letter are not significantly different.

The effect of surface on the residual efficacy of novaluron was not significant until week 2; then it was mostly significant in later bioassays (Figure 2). At week 0, no adult emerged from larvae exposed to 4 ppm on all the tested surfaces. Conversely, emergence was 100% in the control on all surfaces at each exposure period, and thus was not displayed in a panel of Figure 2. The efficacy of novaluron decreased with time after treatment application from week 0 to 16 (Figure 1 and Figure 2). At week 12, the minimum mean adult emergence percentage was observed from metal (17.5%), followed by concrete (28.3%) and wood (40.8%) at 4 ppm concentration (Figure 2). After week 12, the slope of the percentage survival increased, indicating a large reduction in novaluron efficacy.

**Figure 3:**
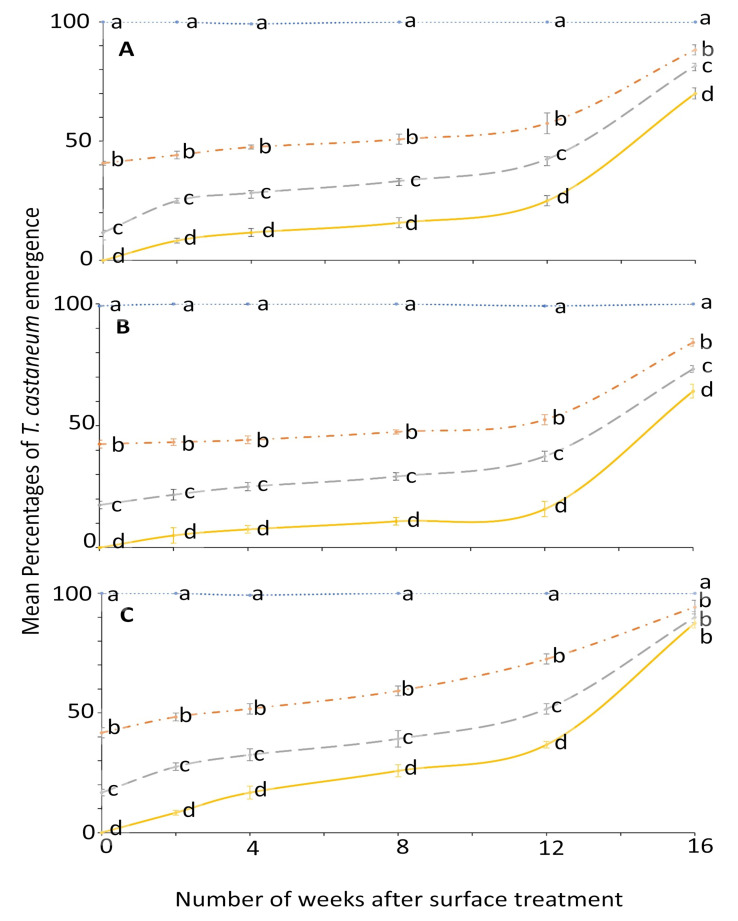
Mean percentages of adults emerging from last-instar *T. castaneum* after exposure to novaluron applied 0–16 weeks previously at concentrations of 4- (solid line), 2- (dashed line), 1- (dashed-dotted line), or 0-ppm (dotted line) onto different surfaces: (**A**) concrete; (**B**) metal; or (**C**) wood. In comparisons among mean emergence percentages from different concentrations tested on a given week after application to a specified surface, designated a–d above, means with the same letter are not significantly different.

**Figure 4:**
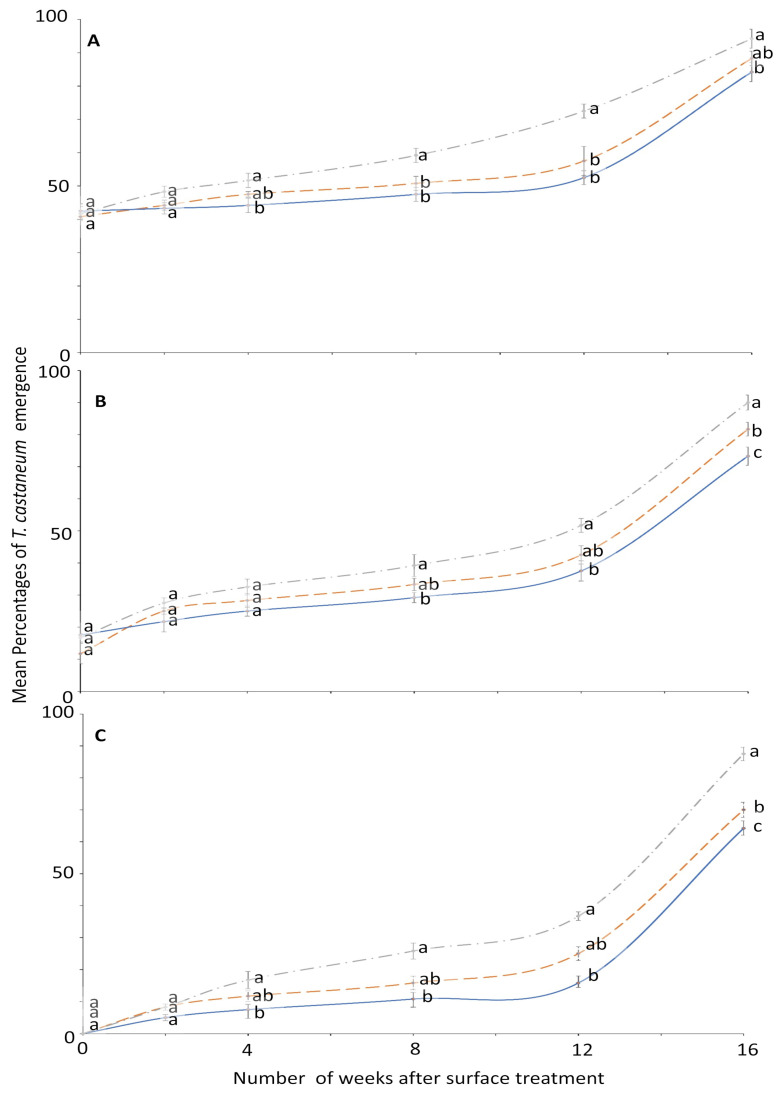
Mean percentages of adults emerging from last-instar *T. castaneum* after exposure to novaluron applied 0–16 weeks previously to different surfaces: concrete (dashed line); metal, (solid line); or wood (dashed-dotted line) at concentrations of (**A**) 1-, (**B**) 2-, or (**C**) 4-ppm. In comparisons among mean emergence percentages from different surfaces on a given week after application at a specified concentration, designated a–d above, percentages with the same letter are not significantly different.

Similarly, the ANOVA for *T. castaneum* showed that all the main effects and their interactions were significant (Figure 3 and Figure 4). The effect of concentration on mean percentage of adult emergence was significant from week 0 to 12; later it became variable (Figure 3).

The percentage emergence means were not significantly different among surfaces until after week 2 (Figure 4). At week 0, no adult emerged from larvae exposed to 4 ppm on all the tested surfaces compared to control where the emergence was 99.2 to 100% (Figure 3). The efficacy of novaluron decreased from week 0 to 16 (Figure 3 and Figure 4). At week 12, the minimum adult emergence percentage was observed for metal (15.8%), followed by concrete (25.0%) and wood (36.7%) at 4 ppm concentration (Figure 4).

**Figure 5:**
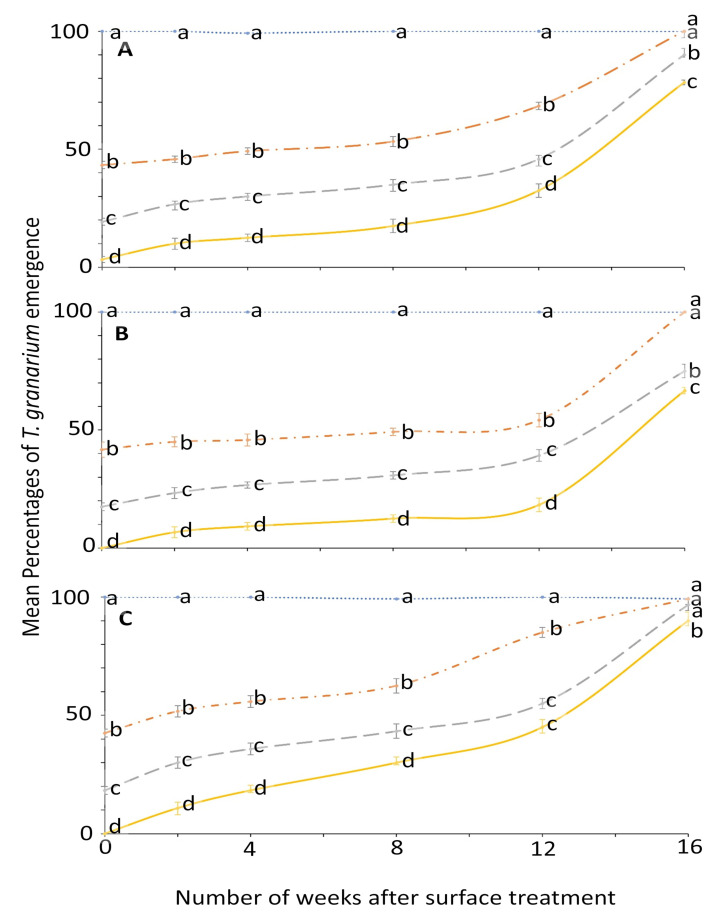
Mean percentages of adults emerging from last-instar *T. granarium* after exposure to novaluron applied 0–16 weeks previously at concentrations of 4- (solid line), 2- (dashed line), 1- (dashed-dotted line), or 0-ppm (dotted line) onto different surfaces: (**A**) concrete; (**B**) metal; or (**C**) wood. In comparisons among mean emergence percentages from different concentrations tested on a given week after application to a specified surface, designated a–d above, means with the same letter are not significantly different.

**Figure 6:**
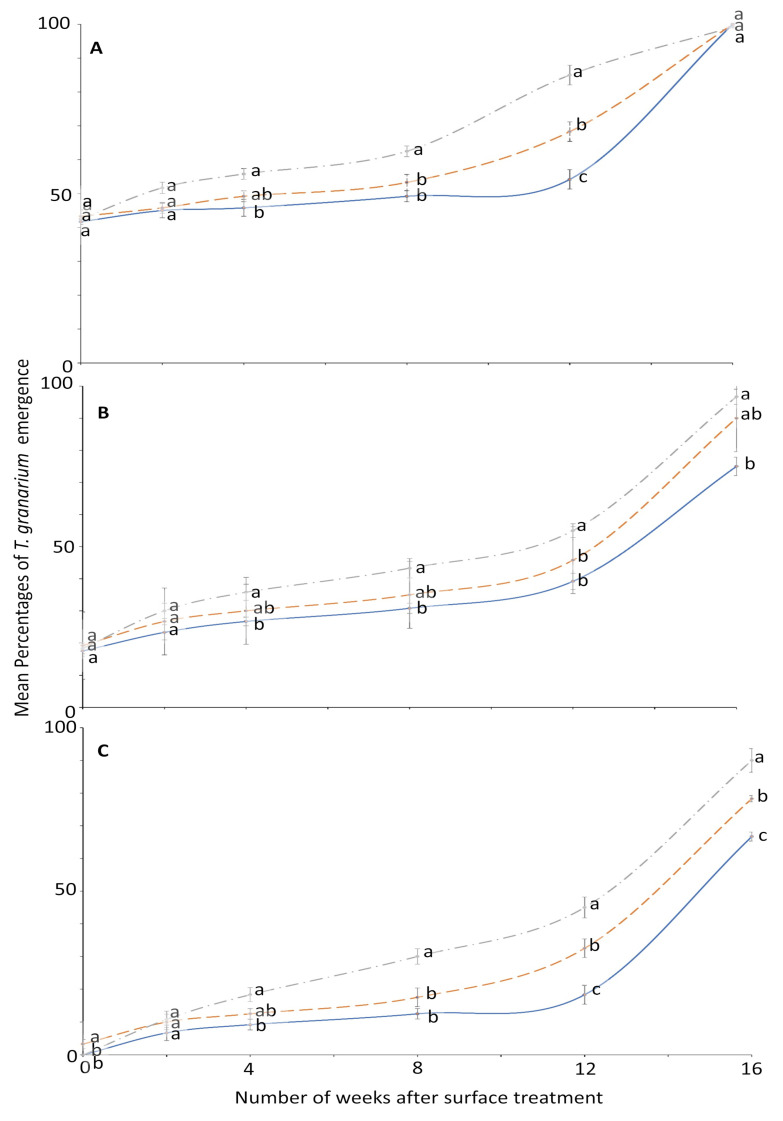
Mean percentages of adults emerging from last-instar *T. granarium* after exposure to novaluron applied 0–16 weeks previously to different surfaces: concrete (dashed line); metal, (solid line); or wood (dashed-dotted line) at concentrations of (**A**) 1-, (**B**) 2-, or (**C**) 4-ppm. In comparisons of mean emergence percentages from surfaces on a given week after application at a specified concentration, designated a–d above, percentages with the same letter are not significantly different.

For *T. granarium*, ANOVA showed that all the main effects and their interactions were significant (Figure 5 and Figure 6). The effect of concentration on adult emergence was significant from week 0 to 8; later it became variable (Figure 5).

The effect of surface on the mean emergence percentage was statistically significant mostly after week 2 (Figure 6). At week 0, no adult emerged from larvae exposed to 4 ppm in metal and wood surface compared to control where the emergence was 100% for all weeks. The efficacy of novaluron decreased with the time after surface treatment from week 0 to 16 (Figure 5 and Figure 6). At week 12, the minimum mean adult emergence percentage at 4 ppm concentration was observed for metal (18.3%), followed by concrete (32.5%) and wood (45.0%) (Figure 6).

## 4. Discussion

The results of this study suggest that the application of novaluron as a surface treatment can significantly reduce the percentage emergence of adult *T. castaneum*, *T. granarium*, and *O. surinamensis* up to 12 weeks after application, depending on the initial application rate. However, statistical significance in comparison to a distilled water control is not the only criterion by which residual efficacy should be assessed. Practical assessments of the efficacy of insecticides as surface treatments depend on several factors, including a manager’s needs for profitability, as well as the type of surface, insecticide and its formulation, exposure period, and insect species [21,37,38,47]. For example, it might be argued that the rate of application should be greater than 0.2 mg/m^2^ to ensure that 95% control of adult emergence is obtained for at least four weeks.

In this study, treatments exhibited more persistence on metal than concrete and wood. Arthur et al. [21] also reported that pyrethroids and insect growth regulators (IGRs) were more persistent on metal surfaces when *T. granarium* larvae were exposed to different treated surfaces. The IGR, methoprene, has similar variation in persistence on various surfaces; with greater percentages of adult emergence on concrete than wood [36,38]. Toews et al. [48] reported greater efficacy of spinosad on concrete than on a steel surface. However, no differences in residual efficacy of pyriproxyfen have been reported when applied to wood, metal and concrete [49]. These variations in residual efficacy might be due to the physical characteristics of the surfaces.

Novaluron provided control of *O. surinamensis*, *T. castaneum*, and *T. granarium*, but other pest species might be present in storage facilities also, some of which may be less susceptible to novaluron. It has been reported that the larvae of *T. castaneum* are more susceptible than *T. confusum* when exposed to surfaces treated with novaluron and methoprene [36]. In another study, *T. variabile* was found to be more tolerant than *T. castaneum* when the larvae of both species were exposed to the combined formulation of novaluron and pyriproxyfen on concrete surfaces [14]. Similarly, *O. surinamensis* was found to be more tolerant than *T. castaneum* when exposed to treatments of surfaces with pyriproxyfen and hydroprene [49]. In a recent study, Yasir et al. [25] found *T. granarium* more tolerant compared to *O. surinamensis* and *T. castaneum* when larvae of these species were exposed to pyriproxyfen-treated grain commodities.

The residual activity of novaluron decreased over time on all tested surfaces, although some efficacy in decreasing emergence by at least 10% remained until after 12 weeks. The general pattern of increase of percentage emergence was essentially independent of the novaluron application rate; as with other pesticides, residual efficacy depends primarily on the rates of degradation [50] and sorption [51] for different substrates. Previous studies with novaluron indicated that it remained effective for four weeks when late instars of *T. castaneum* were exposed to flour treated with 0.2 ppm [32]. Arthur and Hartzer [14] reported that, when the larvae or eggs of *T. castaneum* or *T. variabile* were exposed to concrete surfaces on which combined formulations of novaluron and pyriproxyfen had been applied at their labeled rates, no adults emerged in *T. castaneum* for 16 weeks and emergence did not exceed 25% in *T. variabile* for 0–8 weeks. Similarly, pyriproxyfen has been found to be effective for 12 weeks when the larvae of *T. castaneum*, *T. granarium* and *O. surinamensis* were exposed to treated grain commodities [25] and for 8 weeks when larvae of *T. castaneum* and *O. surinamensis* were exposed to the treated surfaces [49].

One of the purposes of the study was to identify minimum application rates of novaluron that could be useful for insect pest control in small-scale storage facilities where risks from pesticide residues are a concern. In initial trials at week 0, the 0.053, 0.1045, and 0.209 mg/m^2^ application rates were effective for 100% control in two species and allowed a maximum of 3.3% adult emergence in one trial with *T. granarium* at 4 ppm. Consequently, such rates could be considered effective for initial management of infestations of the three insect pest species tested. When managers need protection for longer than four-week periods, the results suggest that higher application rates may be needed to be certain of providing enough protection against buildup of pest populations and reduction of economic profitability.

A rationale for considering novaluron for expanded use as a surface treatment to protect stored products from insect pests is an expected reduction in efficacy of commonly used insecticides due to buildup of insect resistance. However, there may be potential as well for novaluron resistance to develop in stored product insect pests. Insecticide persistence is one of the factors that could contribute to resistance development [52]. Natural variation in susceptibility to novaluron may be another factor, as Cutler et al. [53] suggested in studies with *Leptinotarsa decemlineata* (Say) (Coleoptera: Chrysomelidae) and Parys et al. [54] found in studies with *Lygus lineolaris* (Hemiptera: Miridae). Indeed, field populations of *Tetranychus urticae* Koch (Tetranychidae: Trombidiformes) [55], and *Plutella xylostella* (L.) (Plutellidae: Lepidoptera) [56] have been found that are resistant to etoxazole, which has a similar mode of action as novaluron. These and *Frankliniella occidentalis* (Thysanoptera: Thripidae) have been demonstrated to have achieved resistance through point mutations in *chitin synthase 1* [57]. To reduce the likelihood that stored product pests would begin to exhibit resistance when novaluron is applied to surfaces frequently, application rates may need to be greater 0.209 mg/m^2^.

However, it should be noted that Ishaaya et al. [58] reported no appreciable resistance to novaluron in *Spodoptera littoralis* (Boisduval) (Noctuidae: Lepidoptera), and no cross-resistance in *Bemisia tabaci* (Gennadius) (Aleyrodidae: Hemiptera) resistant to pyriproxyfen. Likewise, there appeared to be no resistance to novaluron in *Aedes aegypti* (L.) (Culicidae: Diptera) in field studies on two different continents [59,60].

Given the limited numbers of insects that have developed resistance until now, novaluron may continue to have relevance as one of several alternatives for growers facing pest populations resistant to neurotoxic insecticides and insect growth regulators if it is used judiciously to avoid development of resistance.

## 5. Conclusions

Novaluron treatments reduced the percentage emergence of *O. surinamensis*, *T. castaneum*, and *T. granarium* for up to 12 weeks on concrete, metal, and wood surfaces. However, it is advisable to further evaluate this product in combination with other inexpensive insecticides that remain suitable for the complete control of these three stored grains pests until resistance overcomes their benefits or those of novaluron. Ultimately a reduced-cost version of novaluron and other chitin-synthesis inhibitors [14,26] may lead to reduced costs of managing stored product insects in increasingly warm, infestation-supporting climates.

## Figures and Tables

**Table 1:** ANOVA for main effects and interactions for adult emergence of *Oryzaephilus surinamensis*, *Tribolium castaneum*, and *Trogoderma granarium* (Error df: 216).

Source	df	*O. surinamensis*		*T. castaneum*		*T. granarium*	
		F	*p*	F	*p*	F	*p*
Week	5	1246.79	<0.01	1043.94	<0.01	1185.98	<0.01
Concentration	3	6380.08	<0.01	6147.63	<0.01	5438.36	<0.01
Surface	2	116.49	<0.01	106.00	<0.01	130.65	<0.01
Week × Concentration	15	147.22	<0.01	129.31	<0.01	140.26	<0.01
Week × Surface	10	9.88	<0.01	9.32	<0.01	11.92	<0.01
Concentration × Surface	6	14.67	<0.01	12.40	<0.01	16.50	<0.01
Week × Concentration × Surface	30	2.83	<0.01	1.90	<0.01	3.79	<0.01

## Data Availability

The data presented in this study are available on request from the first author.
